# Inflammation in Periodontal Disease: Possible Link to Vascular Disease

**DOI:** 10.3389/fphys.2020.609614

**Published:** 2021-01-14

**Authors:** Oindrila Paul, Payal Arora, Michael Mayer, Shampa Chatterjee

**Affiliations:** ^1^Institute for Environmental Medicine, Department of Physiology, University of Pennsylvania School of Medicine, Philadelphia, PA, United States; ^2^Early-Research Oral Care, Colgate-Palmolive Company, Piscataway, NJ, United States; ^3^Department of Radiology, University of Pennsylvania School of Medicine, Philadelphia, PA, United States

**Keywords:** vascular inflammation, cardiovascular disease, dental plaque, risk factors, oxidative stress, antioxidants, NLRP3 inflammasome, periodontal disease (PD)

## Abstract

Inflammation is a well-organized protective response to pathogens and consists of immune cell recruitment into areas of infection. Inflammation either clears pathogens and gets resolved leading to tissue healing or remains predominantly unresolved triggering pathological processes in organs. Periodontal disease (PD) that is initiated by specific bacteria also triggers production of inflammatory mediators. These processes lead to loss of tissue structure and function. Reactive oxygen species and oxidative stress play a role in susceptibility to periodontal pathogenic bacterial infections. Periodontal inflammation is a risk factor for systemic inflammation and eventually cardiovascular disease (CVD). This review discusses the role of inflammation in PD and its two way association with other health conditions such as diabetes and CVD. Some of the mechanisms underpinning the links between inflammation, diabetes, CVD and PD are also discussed. Finally, we review available epidemiological data and other reports to assess possible links between oral health and CVD.

## Introduction

Periodontal disease (PD) is a chronic inflammatory disorder characterized by the destruction of the periodontium, or the supporting tissues of the teeth (gingival tissue, periodontal ligament, and alveolar bone). PD is highly prevalent, and approximately 50% of adults 30 years and older and 70% of adults 65 or older have a form of the disease (Eke et al., 2012). Clinically, the failure to treat PD leads to loss of teeth (Schultz-Haudt et al., 1954; Hajishengallis et al., 2011; Abusleme et al., 2013). Central to PD is dysregulation of the resolution of inflammation, resulting in characteristic chronic and progressive destruction.

Inflammation is a programmed signaling event initiated to protect organisms upon infection and/or injury. In general, infection-injury stimuli lead to release of pathogen or danger associated molecular patterns (PAMPs or DAMPs) followed by their binding to respective receptors in the host cells. Although there are multiple contributing factors to PD, one of the increasingly well-characterized triggers is the colonization of the oral cavity by pathogenic bacteria and their subsequent penetration into local epithelial lining (Darveau, 2010; Abusleme et al., 2013). This initiates an inflammatory cascade characterized by increased expression of various inflammatory mediators and adhesion molecules that collectively mobilize and recruit polymorphonuclear neutrophils (PMN), macrophages, natural killer (NK), dendritic cells (DC) etc. into the affected tissue. Under normal conditions, neutrophils and macrophages phagocytose the microbial organisms after which they undergo apoptosis at the inflamed site (Fox et al., 2010). The clearance of apoptotic cells facilitates a switch from a pro- to an anti-inflammatory macrophage phenotype (Fadok et al., 1998; Michlewska et al., 2009) and initiates the onset of the resolution of inflammation, a coordinated signaling process that restores tissue integrity and function. However, failure to switch off the inflammation cascade once the pathogenic stimulus is removed, leads chronic inflammation (i.e., an uncontrolled inflammatory response that can culminate into damage to the host tissue) and is the hallmark of several inflammatory disorder related pathologies. In PD specifically, the inflammatory response becomes chronic when pathogenic bacteria continue to propagate and cannot be controlled by the acute immune response, resulting in unresolved inflammation, destruction of local bone and soft tissue, and fibrosis (Cochrane, 2008).

Importantly, reports have consistently highlighted a role for periodontal inflammation in acceleration of various vascular pathologies and other systemic implications (Humphrey et al., 2008; Ogrendik, 2013; Ketabi et al., 2016). The destruction of local epithelium by PD pathogens can result in release of local inflammatory mediators from the periodontal pocket into the systemic circulation thus facilitating immune cell recruitment elsewhere. Also, bacteria can either indirectly (within immune cells that have ingested them) or directly circulate in the bloodstream (Lockhart et al., 2009). Therefore, under conditions where there is disposition (e.g., genetic, lifestyle) toward cardiovascular disease (CVD), the bacterial components and systemic inflammatory mediators can potentially accelerate plaque formation. To this point, PD pathogens have been detected in distant tissues and organs, particularly in the cardiovascular system (Okuda et al., 2001; Kozarov et al., 2006; Nakano et al., 2006; Pessi et al., 2013; Moreno et al., 2017). The relationship between PD and systemic diseases such as CVD has been increasingly well-characterized. Importantly, two classic meta-analyses demonstrated the correlation between PD and CVD, highlighting PD as a potential risk factor for CVD processes such as coronary artery disease (Janket et al., 2003; Khader et al., 2004). Additionally, recent evidence suggests a major role for reactive oxygen species (ROS) and proteolytic enzymes (bacteria- and host-derived) in PD and CVD such as atherosclerosis (Chistiakov et al., 2016).

CVD is an umbrella term for a number of linked pathologies, commonly defined as coronary heart disease (CHD), cerebrovascular disease, peripheral arterial disease, rheumatic and congenital heart diseases, and venous thromboembolism (Lara-Pezzi et al., 2012; Mandviwala et al., 2016). The associated risk factors include ethnicity, age, and family history of CVD, dyslipidemia, hypertension, tobacco smoke, excess body weight, physical inactivity, and diabetes mellitus. It is well established that these classic risk factors interact with cellular immune-inflammatory signaling processes to lead to endothelial dysfunction and atheromatous plaque development (Lopez-Candales et al., 2017; Lazzerini et al., 2019). Thus chronic inflammation plays a crucial role in the long-term progression of atherosclerosis. About 35–50% of the world population suffers from periodontitis as reported by World Health Organization (Petersen and Ogawa, 2012); therefore understanding any correlation or link between PD and CVD is a question that has tremendous importance given the high incidences of both diseases. This review summarizes pathophysiology of PD and examines the possibility of its link with CVD.

## Periodontal Disease

The inflammation of tissues in gingivitis and periodontitis is caused by a host of bacteria (Schultz-Haudt et al., 1954). The bacterial species present in the gingival margin are *Porphyromonas gingivalis*, *Treponema denticola*, and *Tannnerella forsythia*, all of which are Gram negative. Also present are Gram positive bacteria like *Streptococcus sanguis*, *Streptococcus oralis*, *Streptococcus mutans*, *Actinomyces naeslundii*, and *Actinomyces odontolyticus (Abusleme et al., 2013)*. This is followed by appearance of secondary bacteria such as *Fusobacterium nucleatum* (Kolenbrander et al., 1989). Aggregates of bacterial colonies form and Gram-positive and Gram-negative bacilli become embedded in the extracellular matrix (Gibbons, 1989). Indeed more than 700 bacterial species have been reported to be detected in dental plaques (Moore, 1987; Gao et al., 2018). Bacterial species normally act as symbiotic communities with the host but shifts of the oral microbiome often associated with “poor” host health can lead to dysbiosis, an imbalance that is responsible for the development of microbe-related PD (Socransky et al., 1998; Darveau, 2010).

**FIGURE 1:**
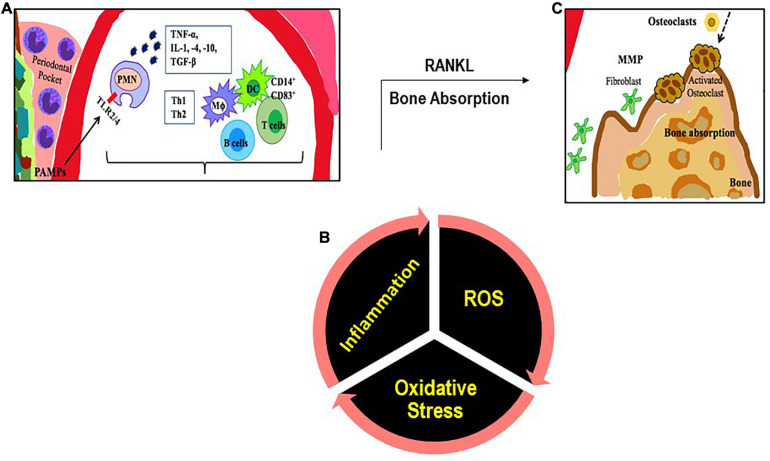
An illustration of the inflammatory immune response with PD. **(A)** PAMPs (like LPS) are recognized by TLRs. The PAMP-TLR interaction triggers a proinflammatory signaling cascade that drives a chemokine and cytokine rich environment into which multiple immune cells [macrophages (MΦ), T and B lymphocytes, dendritic cells (DC)] are recruited. **(B)** Multiple immune cells produce ROS. **(C)** T cells produce the cytokine RANKL that participates in bone resorption.

Several of these bacteria are also present in healthy individuals; however it is their relative abundance due to with poor oral hygiene (Hoare et al., 2019), tobacco consumption etc. that drives the selection and prevalence of pathogenic bacteria in subgingival margin that lead to the onset of periodontitis (Schultz-Haudt et al., 1954; Socransky et al., 1998). Increased oxidative stress with smoking, lifestyle diseases and aging also plays a role; indeed a strong association between oxidative stress and PD has been reported (Chapple and Matthews, 2007). This occurs either due to diminution of antioxidants or an exaggerated inflammatory response post periodontal infection. This is described in the next section. Bacterial plaque formation leads to increases in PAMPs causing a rise in local inflammation, causing increased flow of gingival crevicular fluid (GCF). This in turn provides protein rich nutrients that increase the proliferation of the Gram-negative bacteria. The dental plaque biofilm of bacteria in the periodontal crevice induces clinical signs of inflammation. The progression of PD is driven primarily by the proliferation of *P. gingivalis* which facilitates increase in harmful microbiota. The next step is the secondary bacteria *F. nucleatum’s* role in the subgingival biofilm as this bacterium interacts with other bacterial species found in the biofilm. *F. nucleatum* serves as a bridge between the early colonizers like *Streptococcus* sp. and the late colonizers like *P. gingivalis*. The innate immune response is the recruitment of PMN and the NK cells that is driven by the subgingival bacterial community present in the periodontal pocket. As the microorganisms are abundant, PMN recruitment and phagocytosis is followed by extensive PMN apoptosis or necrosis. A cytokine rich proinflammatory environment consists of tumor necrosis factor (TNF)-α, interleukins (IL)-1, IL-4, IL-10, interferon (IFN-γ) and transforming growth factor (TGF-β). These signaling molecules stimulate the activation of enzymes and transcription factors that in turn recruit more immune cells and degrade the surrounding tissues by maintaining a continual loop of local inflammation (Cekici et al., 2014; Figure 1). This is also accompanied by an adaptive immune response as antigen uptake and processing is carried out by DC and presented to naive T cells. DCs direct CD4+ T cells to differentiate to T-cell subsets such as T helper cells types 1, 2, and 17, and regulatory T cells (Song et al., 2018). CD4+ T cells produce the bone resorption promoting cytokine, Receptor activator of nuclear factor-κB (RANK-L; Tang et al., 2007) leading to bone loss.

## Oxidative Stress in PD and CVD

**FIGURE 2:**
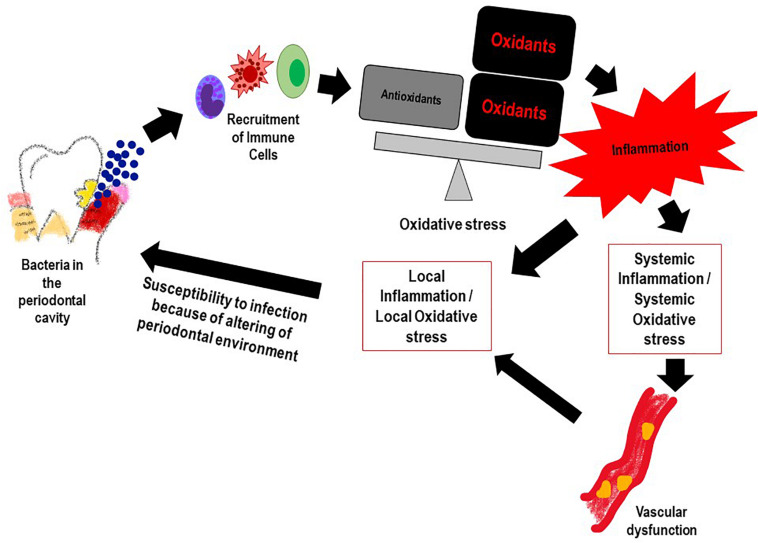
The feed forward mechanism of the “Infection-ROS-Inflammation” triad that seems to accelerate susceptibility to infection, inflammation and oxidative stress.

Reactive oxygen species and the resultant oxidative stress plays an important role in onset and progression of PD (Chapple and Matthews, 2007). This occurs via multiple mechanisms. First, is the ROS production that occurs with periodontal infection, as inflammatory cells are recruited to the infection site with chronic or aggressive periodontitis. Numerous reports have shown that PMN in the population diagnosed with PD generate significantly more ROS (upon stimulation) as compared to PMN of healthy controls (Aboodi et al., 2011; White et al., 2014; Ling et al., 2016). While this is indicative of a hyper-reactive phenotype of neutrophils in the PD affected, it also suggests that high oxidative stress arising from the excessive ROS could increase local gingival oxidative stress which in turn would drive more inflammation. Alteration of the gingival crevicular environment increase susceptibility to periodontal pathogenic bacteria. Second, is the antioxidant status of PD affected individuals; several studies have shown that the low levels of anti-oxidants (that may be associated with high levels of ROS) in the GCF activated the local periodontal inflammation and caused oxidative injury and destruction of the tissue (Tsai et al., 2005; Chapple and Matthews, 2007; Konopka et al., 2007). Figure 2 shows the feed forward loop of infection induced ROS and oxidative stress that in turn drives more inflammation and changes the local gingival tissue environment making it more susceptible to infection. Lifestyle diseases also play a part in this ROS oxidative stress inflammation cascade.

**FIGURE 3:**
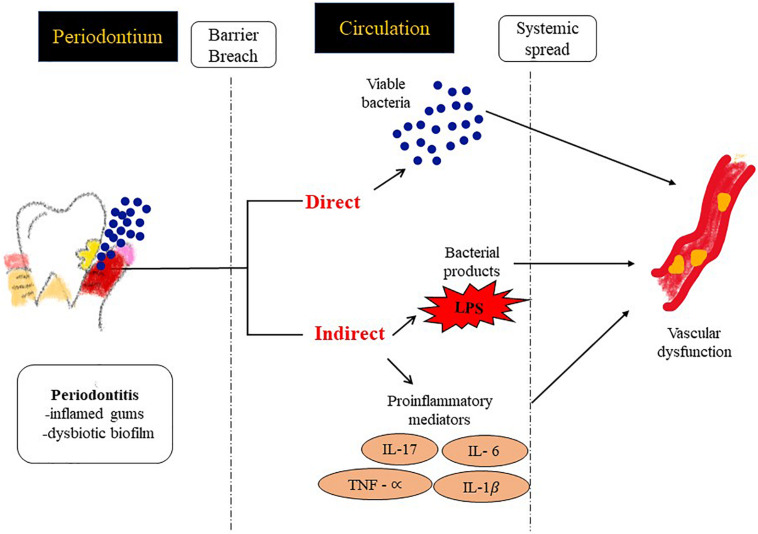
Possible mechanisms by which PD contributes to inflammation at distal sites and thus drives an atherosclerotic phenotype. (1) Direct: vascular infection by periodontal bacterial pathogens and (2) Indirect: facilitating the passage of inflammatory modulators into the systemic circulation.

As is well established, inflammation and oxidative stress are pivotal events that lead to CVD (Mandviwala et al., 2016; Cervantes Gracia et al., 2017) and seem to be the common link between the onset of tissue destruction in periodontitis and systemic inflammation (Wang et al., 2017). Indeed several lifestyle and age related conditions associated with CVD (such as diabetes, hypertension etc.) that lead to high oxidative stress (as assessed by markers of ROS and lipid peroxidation) can also increase susceptibility to PD (Dhadse et al., 2010). When periodontitis susceptible patients are exposed to the bacterial antigen, their main two immune responses in the form of neutrophil recruitment and proteolytic enzymes production further release ROS at the gingival site, thus perpetuating oxidative stress and tissue damage (Scott and Krauss, 2012; Cortes-Vieyra et al., 2016). As periodontitis progresses, periodontal inflammation produces ROS that diffuses into the blood stream (Sobaniec and Sobaniec-Lotowska, 2000; Tomofuji et al., 2007). As a result, various moieties in the blood get oxidized and induce an oxidative stress on other organs via the circulating blood causing circulating oxidative stress (Yagi, 1987; Tomofuji et al., 2007; Figure 3). Thus, it can be inferred that the bacteria present in the periodontal pocket suppress detoxification of ROS by consuming the antioxidants present in the pockets of the oral cavity (Ekuni et al., 2009). The consequence of the lowered levels of antioxidants enables ROS to enter into systemic circulation from the periodontal tissues (Wang et al., 2017).

Regular smoking, diabetes mellitus, insufficient and appropriate nutrition, and aging have all been mentioned as risk factors for both PD and CVD (Yanbaeva et al., 2007; Graves and Kayal, 2008; Dhadse et al., 2010). All the above lifestyle diseases have been known to increase the circulating oxidative stress; indeed increase in levels of malondialdehyde (MDA) and 4-hydroxynonenal (HNE) locally and systemically with PD and with CVD have been observed, thus suggesting an association between local and systemic oxidative stress diseases (Celec et al., 2005; Guentsch et al., 2008; Hendek et al., 2015). Similarly, levels of antioxidants such as SOD and glutathione decrease in the (GCF) and saliva due to smoking in both, healthy individuals and patients with periodontitis (Guentsch et al., 2008; Agnihotri et al., 2009).

## Risk Factors (PD and Vascular Inflammation)

Vascular inflammation involves the onset of a signaling cascade that is triggered by endothelial signaling which leads to increase in cellular adhesions molecules, cytokines and chemokines. This leads to recruitment and adherence of immune cells. The atherogenic process starts with endothelial dysfunction and the accumulation of several plasma low density lipoproteins (LDL) in the subendothelial space. The accumulation of LDL correlates with classical risk factors, such as smoking, hypertension, and metabolic dysregulation in obesity and diabetes mellitus (Gimbrone et al., 2000). As these risk factors are largely associated with PD too, it is reasonable to conclude that common biochemical signaling pathways play a role in vulnerability to both CVD and PD. All the major risk factors associated with PD either activate pathogen initiated inflammation signals (bacteria like *P. gingivalis* and *A. actinomycetemcomitans B. forsythus, P. intermedia, P. micros*, and *F. nucleatum* (Lovegrove, 2004; Saito et al., 2008) or a life style (diabetes mellitus, obesity, aging, smoking, vascular disease) driven inflammatory cascade (Jensen et al., 1991; Grossi and Genco, 1998; Cohen, 2000; Merchant et al., 2003; Humphrey et al., 2008; Hujoel, 2009; Kumar et al., 2011; Nakamura et al., 2011; Ozcaka et al., 2011). Nutrition and oral health are closely linked. This is because oxidative stress and antioxidant balance which drives ROS induced inflammation signals, can be regulated by diets rich in antioxidants (Muniz et al., 2015). Diets that lead to obesity such as high carbohydrates and sugars have been implicated in dental caries and PD as these drive plaque formation and accelerate inflammation thus causing dental tissue oxidative damage and decay (Hujoel, 2009). Changes in dietary intake influence the extent of PD. As seen in the dietary intake of adolescents between 11 and 18 years old, the decrease in consumption of raw fruits and non-potato vegetables with concomitant increase in uptake of soft drinks, led to increased PD (Chaffee and Weston, 2010).

Indeed when all these risk factors are controlled, both PD and CVD show improvement (Table 1; Higashi et al., 2008; Bokhari et al., 2012; Montenegro et al., 2019; Lobo et al., 2020).

**TABLE 1:** Clinical trials investigating relationship between periodontal disease and cardiovascular disease.

**Citation**	**Aims**	**Patients**	**Cohorts**	**Outcomes**
Elter et al., 2006	Effect of periodontal treatment on endothelium-dependent flow-mediated dilation and serum inflammatory biomarkers in patients with PD	Twent-two male patients with PD	All patients underwent periodontal treatment (scaling and root planning, periodontal flap surgery if indicated, and extraction of hopeless teeth)	Periodontal treatment resulted in significant improvements in flow-mediated dilation and decreased serum IL-6 (and a trend toward reduction in CRP)
D’Aiuto et al., 2006	Effects of intensive periodontal treatment on serum inflammatory biomarkers, serum lipid levels, and blood pressure in patients with PD	Forty patients with severe PD	- Experimental group: subject to intensive periodontal treatment (defined as standard treatment plus adjunctive use of a locally delivered antimicrobial) - Control group: standard periodontal treatment (scaling and root planning)	Intensive periodontal treatment resulted in significant reduction in IL-6, CRP, total cholesterol, and systolic blood pressure at 2-month follow-up
Tonetti et al., 2007	To investigate the effects of periodontal treatment on parameters of endothelial function in patients with PD	Hundred and twnety patients with severe PD	- Experimental group: subject to intensive PD treatment - Control group: subject to community-based periodontal care	Intensive periodontal treatment resulted in reduced brachial artery flow, reduced levels of CRP and IL-6, and elevated endothelial-activation markers.- Intensive treatment was associated with reduced indexes of periodontal disease severity and significantly better endothelial function at 6 month follow-up
Higashi et al., 2008	To evaluate endothelial function in patients with hypertension and PD	Sixty-four patients with hypertension (26 with PD and 38 without PD)	- Experimental group: subject to periodontal treatment - Control group: subject to community-based periodontal care	Periodontal treatment resulted in decreased serum CRP and IL-6 at 24-week follow-up Periodontal treatment resulted in reduced acetylcholine-stimulated vasodilation Delivery of a nitric oxide synthase inhibitor before and after PD treatment resulted in similar acetylcholine-stimulated vasodilation, suggesting role of nitric oxide bioavailability in mechanism of endothelial dysfunction in patients with PD
Higashi et al., 2009	To evaluate endothelial function and the effects of periodontal treatment on patients with CAD and PD	101 patients with CAD (48 with PD and 53 without PD)	- Experimental group: subject to periodontal treatment - Control group: subject to community-based periodontal care	- Periodontal treatment resulted in decreased serum CRP and IL-6 at follow-up - Periodontal treatment resulted in reduced acetylcholine-stimulated vasodilation - Delivery of a nitric oxide synthase inhibitor before and after PD treatment resulted in similar acetylcholine-stimulated vasodilation, suggesting role of nitric oxide bioavailability in mechanism of endothelial dysfunction in patients with PD
Offenbacher et al., 2009	Impact of periodontal treatment on serum CRP levels, clinical PD parameters, and cardiovascular endpoints in patients with PD	Three hundred three patients with PD and previous history of CVD	- Experimental group: subject to periodontal treatment (scaling and root planning - Control group: not subject to periodontal treatment	Periodontal treatment resulted in a significant improvement in PD status at 6 months as assessed by reduction of probing depth, but no difference in attachment levels, bleeding upon probing, or extent of subgingival calculus Periodontal treatment resulted in significant decrease of the odds of being in the high-risk (>3 mg/L) CRP group at 6 months, with obesity nullifying such effect
Vidal et al., 2009	Effects of non-surgical PD treatment on plasma levels of inflammatory markers (interleukin [IL]-6, C-reactive protein [CRP], and fibrinogen) in patients with severe PD and refractory arterial hypertension	Twenty-two patients with severe PD and refractory arterial hypertension	- Experimental group: subject to periodontal treatment at start of trial - Control group: subject to periodontal treatment delayed 3-months from start of trial	Periodontal treatment resulted in significant reduction in markers of PD severity (probing, probing depth, and clinical attachment loss) Periodontal treatment resulted in significant reduction of fibrinogen, CRP, and IL-6
Wehmeyer et al., 2013	Impact of periodontal treatment PD parameters and inflammatory biomarkers in patients with end-stage renal disease and PD	Three hundred forty-two dialysis patients with moderate/severe chronic PD	- Experimental group: subject to intensive periodontal treatment - Control group: subject to intensive periodontal treatment following study completion at 6 months	Intensive periodontal treatment resulted in significantly improved measures of periodontal health at 3 months, but only PD remained significantly different at 6 months No significant difference between the groups inflammatory biomarkers (serum albumin or high-sensitivity interleukin 6) at any time point
Bokhari et al., 2012	Effect of non-surgical periodontal treatment on systemic C-reactive protein, fibrinogen and white blood cells in coronary heart disease patients with PD	Three hundred seventeen patientswith angiographically proven coronary heart disease	- Experimental group: subject to on-surgical periodontal treatment (scaling, root planning and oral hygiene instructions) - Control group: not subject to periodontal treatment	Non−surgical periodontal treatment resulted in significant reduction of systemic levels of inflammatory markers (CRP, fibrinogen and WBCs) at 2-month follow-up
Caula et al., 2014	Influence of non−surgical mechanical PD treatment on c-reactive protein serum level, erythrocyte sedimentation rate (ESR), and lipid profile in patients with severe chronic periodontitis	Sixty-four patients with severe chronic PD	- Experimental group: Began non-surgical PD treatment - Control group: Withheld PD treatment during study period	PD treatment resulted in a significant reduction of ESR and triglycerides at 2 months PD treatment resulted in significant reduction in median values of C−reactive protein, ESR, total cholesterol, and triglycerides after 6 months
Zhou et al., 2017	To investigate if intensive periodontal treatment can lower blood pressure levels and endothelial microparticles (EMPs) in patients with prehypertension and PD without antihypertensive medication	Hundred and seven patients with prehypertension and PD	- Experimental group: Subject to intensive periodontal treatment - Control group: Subject to community−based periodontal care	Intensive periodontal treatment resulted in reduced clinical PD parameters, reduced systolic and diastolic blood pressures, and reduced endothelial microparticles
Montenegro et al., 2019	To assess the effect of periodontal treatment on clinical PD parameters and levels of cardiovascular risk biomarkers in stable coronary artery disease (CAD) patients (CRP, glycated hemoglobin, lipids, IL−1β, IL−6, IL−8, IL−10, IFN−γ and TNF−α)	88 patients with stable coronary artery disease and periodontitis	- Experimental group: Subject to non-surgical periodontal treatment - Control group: Subject to one session of plaque removal	Periodontal treatment resulted in significantly better periodontal parameters after 3 months without significant differences in blood biomarkers In patients with baseline high levels of CRP, periodontal treatment resulted in lower levels of CRP, IL−6 and IL−8
Lobo et al., 2020	To investigate the impact of periodontal treatment on the endothelial function of patients with a recent ST-segment elevation myocardial infarction (STEMI), specifically looking at variation of flow-mediated vasodilation (FMD) in the brachial artery, inflammatory biomarkers, and adverse CVD events	Forty-eight patients with PD and with recent admission for STEMI	- Experimental group: subject to periodontal treatment within 2 weeks of STEMI - Control group: not subject to periodontal treatment	Periodontal treatment significantly improved endothelial function of the brachial artery without adverse clinical effects over a period of 6 months Inflammatory biomarkers and cardiovascular events were not significantly different between both groups

## PD and CVD: Is There a Link?

It is not clear if there is a direct and common thread between PD and CVD; however the fact that people with PD have a two or three times higher risk of a cardiovascular event (stroke, heart attack etc.) seems to point to a cluster of shared risk factors between the two (Sanz et al., 2020). The “inflammation” link seems to be a key contributor to both.

For instance, when infected with *P. gingivalis*, the host innate immune system responds by activating inflammation consisting of the NLRP3 inflammasome (pro-inflammatory IL-1β, IL-18) (Lamkanfi and Dixit, 2009; Xue et al., 2015). Patients with chronic PD and aggressive PD expressed significantly higher levels of NLRP3 in gingiva (Xue et al., 2015; Ran et al., 2017). In wild type mice with *P. gingivalis* infection, the increase in expression of NLRP3 inflammation cascade in the gingival tissue was matched with a concomitant increase in caspase-1 activity in the macrophages found in peritoneum; this was not observed in NLRP3 deficient mice (Yamaguchi et al., 2017). This suggests that the NLRP3 inflammasome activated in periodontitis has effects on the systemic organs. NLRP3 inflammasome has also been shown to be highly expressed and activated with systemic vascular disease (Satoh et al., 2014) although it is not clear whether NLRP3 from *P. gingivalis* (Yamaguchi et al., 2017) is directly involved. However, excess ROS, glucose, ATP, ceramides, sphingosine, cholesterol crystals, uric acid and oxidized LDL (all of which are associated with CVD) have been known to activate NLRP3 inflammasome (Duewell et al., 2010; Jiang et al., 2012; Luheshi et al., 2012).

Epidemiologic data till date that suggest an association between PD and CVD (Humphrey et al., 2008; Blaizot et al., 2009; Tonetti, 2009) have monitored PD via indices of clinical attachment level, pocket depth, bleeding on probing and decayed-missing-filled teeth and CVD by degree and number of obstructed coronary arteries, observed an association between PD and CVDs (Ketabi et al., 2016). Table 1 shows the clinical trials which investigated the relationship between the two diseases. While risk factors as discussed in the earlier section play a crucial role in the onset of CVD, increasing number of CVD patients do not harbor the classical risk factors. Low grade infection such as in periodontal infection could be a potential cause for CVD in these cases; indeed several studies show that PD as a risk factor for CVD and, in particular, atherosclerosis (Bartova et al., 2014; Toregeani et al., 2016).

Potential links between PD and CVD could be via two mechanisms (Figure 3).

1. Systemic Inflammation: Systemic inflammatory markers such as C-reactive protein (CRP), IL-6 etc. have shown direct correlation with specific indices of CVD such as carotid-intima media thickness, or MI (myocardial infarct size) (Ali et al., 2006). Chronic periodontal infection is characterized by elevation of CRP and inflammatory cytokines in the systemic circulation (Loos et al., 2000), so it is possible that systemic inflammation in patients with PD can potentially accelerate endothelial dysfunction, plaque buildup and CHD events.
2. Vascular Infection: There have been reports that identify bacterial species in blood after dental procedures suggesting gingiva as a portal via which oral bacterial pathogens can enter the systemic circulation (Bahrani-Mougeot et al., 2008; Lockhart et al., 2009). As a result bacteremia of dental origin seems to play a role in the appearance of bacterial endocarditis (Mang-de la Rosa et al., 2014) and periodontal bacterial components colonize human atheromatous plaques (Haraszthy et al., 2000; Fiehn et al., 2005).

## Conclusion

While both PD and CVD have manifestations of classic inflammation, a causative link between them has not been established. However, oxidative stress arising from lifestyle diseases play a crucial role in progression of both PD and CVD, indicating that host influence in terms of an imbalance between ROS production and endogenous antioxidant levels can increase susceptibility in individuals.

Understanding how oxidative stress and inflammation overlap in PD and CVD for high risk and older populations is of great public health importance because of the high prevalence of PD. Although both these pathologies arise from the same risk factors and show a similar systemic inflammation profile, it is not clear how these diseases intersect. Therefore it cannot be concluded that therapeutic periodontal interventions can prevent heart disease or stroke. Nevertheless, controlling the overall inflammation status by implementing a good periodontal maintenance program could presumably control the progression of CVD in periodontitis patients.

## Author Contributions

OP drafted the article and prepared the figures. PA drafted the article. MM drafted the article and prepared the table. SC developed the concept and outline, drafted the article, and formalized the final version. All authors approved the submission.

## Conflict of Interest

The authors declare that the research was conducted in the absence of any commercial or financial relationships that could be construed as a potential conflict of interest.

## References

[B1] Aboodi G. M. Goldberg M. B. Glogauer M. (2011). Refractory periodontitis population characterized by a hyperactive oral neutrophil phenotype. *J. Periodontol.* 82 726–733. 10.1902/jop.2010.100508 21080789

[B2] Abusleme L. Dupuy A. K. Dutzan N. Silva N. Burleson J. A. Strausbaugh L. D. (2013). The subgingival microbiome in health and periodontitis and its relationship with community biomass and inflammation. *ISME J.* 7 1016–1025. 10.1038/ismej.2012.174 23303375 PMC3635234

[B3] Agnihotri R. Pandurang P. Kamath S. U. Goyal R. Ballal S. Shanbhogue A. Y. (2009). Association of cigarette smoking with superoxide dismutase enzyme levels in subjects with chronic periodontitis. *J. Periodontol.* 80 657–662. 10.1902/jop.2009.080545 19335086

[B4] Ali Y. S. Rembold K. E. Weaver B. Wills M. B. Tatar S. Ayers C. R. (2006). Prediction of major adverse cardiovascular events by age-normalized carotid intimal medial thickness. *Atherosclerosis* 187 186–190. 10.1016/j.atherosclerosis.2005.09.003 16233899

[B5] Bahrani-Mougeot F. K. Paster B. J. Coleman S. Ashar J. Barbuto S. Lockhart P. B. (2008). Diverse and novel oral bacterial species in blood following dental procedures. *J. Clin. Microbiol.* 46 2129–2132. 10.1128/jcm.02004-07 18434561 PMC2446827

[B6] Bartova J. Sommerova P. Lyuya-Mi Y. Mysak J. Prochazkova J. Duskova J. (2014). Periodontitis as a risk factor of atherosclerosis. *J. Immunol. Res.* 2014:636893. 10.1155/2014/636893 PMC3987959 24741613

[B7] Blaizot A. Vergnes J. N. Nuwwareh S. Amar J. Sixou M. (2009). Periodontal diseases and cardiovascular events: meta-analysis of observational studies. *Int. Dent. J.* 59 197–209. 19774803

[B8] Bokhari S. A. Khan A. A. Butt A. K. Azhar M. Hanif M. Izhar M. (2012). Non-surgical periodontal therapy reduces coronary heart disease risk markers: a randomized controlled trial. *J. Clin. Periodontol.* 39 1065–1074. 10.1111/j.1600-051x.2012.01942.x 22966824

[B9] Caula A. L. Lira-Junior R. Tinoco E. M. Fischer R. G. (2014). The effect of periodontal therapy on cardiovascular risk markers: a 6-month randomized clinical trial. *J. Clin. Periodontol.* 41 875–882. 10.1111/jcpe.12290 25041550

[B10] Cekici A. Kantarci A. Hasturk H. Van Dyke T. E. (2014). Inflammatory and immune pathways in the pathogenesis of periodontal disease. *Periodontology* 2000 57–80. 10.1111/prd.12002 24320956 PMC4500791

[B11] Celec P. Hodosy J. Celecova V. Vodrazka J. Cervenka T. Halcak L. (2005). Salivary thiobarbituric acid reacting substances and malondialdehyde–their relationship to reported smoking and to parodontal status described by the papillary bleeding index. *Dis. Markers* 21 133–137. 10.1155/2005/693437 16276007 PMC3850631

[B12] Cervantes Gracia K. Llanas-Cornejo D. Husi H. (2017). CVD and oxidative stress. *J. Clin. Med.* 6:22. 10.3390/jcm6020022 28230726 PMC5332926

[B13] Chaffee B. W. Weston S. J. (2010). Association between chronic periodontal disease and obesity: a systematic review and meta-analysis. *J. Periodontol.* 81 1708–1724. 10.1902/jop.2010.100321 20722533 PMC3187554

[B14] Chapple I. L. Matthews J. B. (2007). The role of reactive oxygen and antioxidant species in periodontal tissue destruction. *Periodontology* 2000 160–232. 10.1111/j.1600-0757.2006.00178.x 17214840

[B15] Chistiakov D. A. Orekhov A. N. Bobryshev Y. V. (2016). Links between atherosclerotic and periodontal disease. *Exp. Mol. Pathol.* 100 220–235. 10.1016/j.yexmp.2016.01.006 26777261

[B16] Cochrane (2008). New cochrane systematic reviews — cochrane oral health group. *J. Evid. Based Dent. Pract.* 8 258–260. 10.1016/j.jebdp.2008.09.001

[B17] Cohen D. W. (2000). Periodontal medicine in the next millennium. *Intl. J. Periodontics Restorative Dent.* 20 6–7. 11203549

[B18] Cortes-Vieyra R. Rosales C. Uribe-Querol E. (2016). Neutrophil functions in periodontal homeostasis. *J. Immunol. Res.* 2016:1396106. 10.1155/2016/1396106 PMC4785262 27019855

[B19] D’Aiuto F. Parkar M. Nibali L. Suvan J. Lessem J. Tonetti M. S. (2006). Periodontal infections cause changes in traditional and novel cardiovascular risk factors: results from a randomized controlled clinical trial. *Am. Heart. J.* 151 977–984. 10.1016/j.ahj.2005.06.018 16644317

[B20] Darveau R. P. (2010). Periodontitis: a polymicrobial disruption of host homeostasis. *Nat. Rev. Microbiol.* 8 481–490. 10.1038/nrmicro2337 20514045

[B21] Dhadse P. Gattani D. Mishra R. (2010). The link between periodontal disease and cardiovascular disease: how far we have come in last two decades? *J. Indian Soc. Periodontol.* 14 148–154. 10.4103/0972-124x.75908 21760667 PMC3100856

[B22] Duewell P. Kono H. Rayner K. J. Sirois C. M. Vladimer G. Bauernfeind F. G. (2010). NLRP3 inflammasomes are required for atherogenesis and activated by cholesterol crystals. *Nature* 464 1357–1361. 10.1038/nature08938 20428172 PMC2946640

[B23] Eke P. I. Dye B. A. Wei L. Thornton-Evans G. O. Genco R. J. (2012). Prevalence of periodontitis in adults in the United States: 2009 and 2010. *J. Dent. Res.* 91 914–920. 10.1177/0022034512457373 22935673

[B24] Ekuni D. Tomofuji T. Sanbe T. Irie K. Azuma T. Maruyama T. (2009). Periodontitis-induced lipid peroxidation in rat descending aorta is involved in the initiation of atherosclerosis. *J. Periodontal Res.* 44 434–442. 10.1111/j.1600-0765.2008.01122.x 19210335

[B25] Elter J. R. Hinderliter A. L. Offenbacher S. Beck J. D. Caughey M. Brodala N. (2006). The effects of periodontal therapy on vascular endothelial function: a pilot trial. *Am. Heart. J.* 151:47. 10.1016/j.ahj.2005.10.002 16368290

[B26] Fadok V. A. Bratton D. L. Konowal A. Freed P. W. Westcott J. Y. Henson P. M. (1998). Macrophages that have ingested apoptotic cells in vitro inhibit proinflammatory cytokine production through autocrine/paracrine mechanisms involving TGF-beta. PGE2, and PAF. *J. Clin. Invest.* 101 890–898. 10.1172/jci1112 9466984 PMC508637

[B27] Fiehn N. E. Larsen T. Christiansen N. Holmstrup P. Schroeder T. V. (2005). Identification of periodontal pathogens in atherosclerotic vessels. *J. Periodontol.* 76 731–736. 10.1902/jop.2005.76.5.731 15898933

[B28] Fox S. Leitch A. E. Duffin R. Haslett C. Rossi A. G. (2010). Neutrophil apoptosis: relevance to the innate immune response and inflammatory disease. *J. Innate Immunity* 2 216–227. 10.1159/000284367 20375550 PMC2956014

[B29] Gao L. Xu T. Huang G. Jiang S. Gu Y. Chen F. (2018). Oral microbiomes: more and more importance in oral cavity and whole body. *Protein Cell* 9 488–500. 10.1007/s13238-018-0548-1 29736705 PMC5960472

[B30] Gibbons R. J. (1989). Bacterial adhesion to oral tissues: a model for infectious diseases. *J. Dent. Res.* 68 750–760. 10.1177/00220345890680050101 2654229

[B31] Gimbrone M. A. Jr. Topper J. N. Nagel T. Anderson K. R. Garcia-Cardena G. (2000). Endothelial dysfunction, hemodynamic forces, and atherogenesis. *Ann. N. Y. Acad. Sci.* 902 230–239. 10.1111/j.1749-6632.2000.tb06318.x 10865843

[B32] Graves D. T. Kayal R. A. (2008). Diabetic complications and dysregulated innate immunity. *Front. Biosci.* 13 1227–1239. 10.2741/2757 17981625 PMC3130196

[B33] Grossi S. G. Genco R. J. (1998). Periodontal disease and diabetes mellitus: a two-way relationship. *Ann. Periodontol.* 3 51–61. 10.1902/annals.1998.3.1.51 9722690

[B34] Guentsch A. Preshaw P. M. Bremer-Streck S. Klinger G. Glockmann E. Sigusch B. W. (2008). Lipid peroxidation and antioxidant activity in saliva of periodontitis patients: effect of smoking and periodontal treatment. *Clin. Oral Investig.* 12 345–352. 10.1007/s00784-008-0202-z 18509684

[B35] Hajishengallis G. Liang S. Payne M. A. Hashim A. Jotwani R. Eskan M. A. (2011). Low-abundance biofilm species orchestrates inflammatory periodontal disease through the commensal microbiota and complement. *Cell Host Microbe* 10 497–506. 10.1016/j.chom.2011.10.006 22036469 PMC3221781

[B36] Haraszthy V. I. Zambon J. J. Trevisan M. Zeid M. Genco R. J. (2000). Identification of periodontal pathogens in atheromatous plaques. *J. Periodontol.* 71 1554–1560. 10.1902/jop.2000.71.10.1554 11063387

[B37] Hendek M. K. Erdemir E. O. Kisa U. Ozcan G. (2015). Effect of initial periodontal therapy on oxidative stress markers in gingival crevicular fluid, saliva, and serum in smokers and non-smokers with chronic periodontitis. *J. Periodontol.* 86 273–282. 10.1902/jop.2014.140338 25325515

[B38] Higashi Y. Goto C. Hidaka T. Soga J. Nakamura S. Fujii Y. (2009). Oral infection-inflammatory pathway, periodontitis, is a risk factor for endothelial dysfunction in patients with coronary artery disease. *Atherosclerosis* 206 604–610. 10.1016/j.atherosclerosis.2009.03.037 19410250

[B39] Higashi Y. Goto C. Jitsuiki D. Umemura T. Nishioka K. Hidaka T. (2008). Periodontal infection is associated with endothelial dysfunction in healthy subjects and hypertensive patients. *Hypertension* 51 446–453. 10.1161/hypertensionaha.107.101535 18039979

[B40] Hoare A. Soto C. Rojas-Celis V. Bravo D. (2019). Chronic Inflammation as a Link between periodontitis and carcinogenesis. *Mediators Inflamm.* 2019:1029857. 10.1155/2019/1029857 PMC6458883 31049022

[B41] Hujoel P. (2009). Dietary carbohydrates and dental-systemic diseases. *J. Dent. Res.* 88 490–502. 10.1177/0022034509337700 19587153

[B42] Humphrey L. L. Fu R. Buckley D. I. Freeman M. Helfand M. (2008). Periodontal disease and coronary heart disease incidence: a systematic review and meta-analysis. *J. Gen. Intern. Med.* 23 2079–2086. 10.1007/s11606-008-0787-6 18807098 PMC2596495

[B43] Janket S. J. Baird A. E. Chuang S. K. Jones J. A. (2003). Meta-analysis of periodontal disease and risk of coronary heart disease and stroke. *Oral Surg. Oral Med. Oral Pathol. Oral Radiol. Endod.* 95 559–569. 10.1067/moe.2003.107 12738947

[B44] Jensen J. A. Goodson W. H. Hopf H. W. Hunt T. K. (1991). Cigarette smoking decreases tissue oxygen. *Arch. Surg.* 126 1131–1134. 10.1001/archsurg.1991.01410330093013 1929845

[B45] Jiang Y. Wang M. Huang K. Zhang Z. Shao N. Zhang Y. (2012). Oxidized low-density lipoprotein induces secretion of interleukin-1beta by macrophages via reactive oxygen species-dependent NLRP3 inflammasome activation. *Biochem. Biophys. Res. Commun.* 425 121–126. 10.1016/j.bbrc.2012.07.011 22796220

[B46] Ketabi M. Meybodi F. R. Asgari M. R. (2016). The association between periodontal disease parameters and severity of atherosclerosis. *Dent. Res. J.* 13 250–255. 10.4103/1735-3327.182185 27274346 PMC4878210

[B47] Khader Y. S. Albashaireh Z. S. Alomari M. A. (2004). Periodontal diseases and the risk of coronary heart and cerebrovascular diseases: a meta-analysis. *J. Periodontol.* 75 1046–1053. 10.1902/jop.2004.75.8.1046 15455730

[B48] Kolenbrander P. E. Andersen R. N. Moore L. V. (1989). Coaggregation of *Fusobacterium nucleatum*, *Selenomonas flueggei*, *Selenomonas infelix*, *Selenomonas noxia*, and selenomonas sputigena with strains from 11 genera of oral bacteria. *Infect. Immun.* 57 3194–3203. 10.1128/iai.57.10.3194-3203.1989 2777378 PMC260789

[B49] Konopka T. Krol K. Kopec W. Gerber H. (2007). Total antioxidant status and 8-hydroxy-2’-deoxyguanosine levels in gingival and peripheral blood of periodontitis patients. *Arch. Immunol. Ther. Exp.* 55 417–422. 10.1007/s00005-007-0047-1 18060366 PMC2766448

[B50] Kozarov E. Sweier D. Shelburne C. Progulske-Fox A. Lopatin D. (2006). Detection of bacterial DNA in atheromatous plaques by quantitative PCR. *Microbes Infect.* 8 687–693. 10.1016/j.micinf.2005.09.004 16513386

[B51] Kumar P. S. Matthews C. R. Joshi V. De Jager M. Aspiras M. (2011). Tobacco smoking affects bacterial acquisition and colonization in oral biofilms. *Infect. Immun.* 79 4730–4738. 10.1128/iai.05371-11 21859855 PMC3257914

[B52] Lamkanfi M. Dixit V. M. (2009). Inflammasomes: guardians of cytosolic sanctity. *Immunol. Rev.* 227 95–105. 10.1111/j.1600-065x.2008.00730.x 19120479

[B53] Lara-Pezzi E. Dopazo A. Manzanares M. (2012). Understanding cardiovascular disease: a journey through the genome (and what we found there). *Dis. Models Mech.* 5 434–443. 10.1242/dmm.009787 22730474 PMC3380707

[B54] Lazzerini P. E. Hamilton R. M. Boutjdir M. (2019). Editorial: cardioimmunology: inflammation and immunity in cardiovascular disease. *Front. Cardiovasc. Med.* 6:181. 10.3389/fcvm.2019.00181 PMC6901670 31850376

[B55] Ling M. R. Chapple I. L. Matthews J. B. (2016). Neutrophil superoxide release and plasma C-reactive protein levels pre- and post-periodontal therapy. *J. Clin. Periodontol.* 43 652–658. 10.1111/jcpe.12575 27168055

[B56] Lobo M. G. Schmidt M. M. Lopes R. D. Dipp T. Feijo I. P. Schmidt K. E. S. (2020). Treating periodontal disease in patients with myocardial infarction: a randomized clinical trial. *Eur. J. Intern. Med.* 71 76–80. 10.1016/j.ejim.2019.08.012 31810741

[B57] Lockhart P. B. Brennan M. T. Thornhill M. Michalowicz B. S. Noll J. Bahrani-Mougeot F. K. (2009). Poor oral hygiene as a risk factor for infective endocarditis-related bacteremia. *J. Am. Dent. Assoc.* 140 1238–1244. 10.14219/jada.archive.2009.0046 19797553 PMC2770162

[B58] Loos B. G. Craandijk J. Hoek F. J. Wertheim-Van Dillen P. M. Van Der Velden U. (2000). Elevation of systemic markers related to cardiovascular diseases in the peripheral blood of periodontitis patients. *J. Periodontol.* 71 1528–1534. 10.1902/jop.2000.71.10.1528 11063384

[B59] Lopez-Candales A. Hernandez Burgos P. M. Hernandez-Suarez D. F. Harris D. (2017). Linking chronic inflammation with cardiovascular disease: from normal aging to the metabolic syndrome. *J. Nat. Sci.* 3:e341. PMC5488800 28670620

[B60] Lovegrove J. M. (2004). Dental plaque revisited: bacteria associated with periodontal disease. *J. N. Z. Soc. Periodontol.* 2004 7–21. 15143484

[B61] Luheshi N. M. Giles J. A. Lopez-Castejon G. Brough D. (2012). Sphingosine regulates the NLRP3-inflammasome and IL-1beta release from macrophages. *Eur. J. Immunol.* 42 716–725. 10.1002/eji.201142079 22105559 PMC3491674

[B62] Mandviwala T. Khalid U. Deswal A. (2016). Obesity and cardiovascular disease: a risk factor or a risk marker? *Curr. Atheroscler. Rep.* 18:21. 10.1007/s11883-016-0575-4 26973130

[B63] Mang-de la Rosa M. R. Castellanos-Cosano L. Romero-Perez M. J. Cutando A. (2014). The bacteremia of dental origin and its implications in the appearance of bacterial endocarditis. *Med. Oral Patol. Oral Cir. Bucal* 19 e67–e74. 24121925 10.4317/medoral.19562 PMC3909435

[B64] Merchant A. T. Pitiphat W. Ahmed B. Kawachi I. Joshipura K. (2003). A prospective study of social support, anger expression and risk of periodontitis in men. *J. Am. Dent. Assoc.* 134 1591–1596. 10.14219/jada.archive.2003.0104 14719755

[B65] Michlewska S. Dransfield I. Megson I. L. Rossi A. G. (2009). Macrophage phagocytosis of apoptotic neutrophils is critically regulated by the opposing actions of pro-inflammatory and anti-inflammatory agents: key role for TNF-alpha. *FASEB J.* 23 844–854. 10.1096/fj.08-121228 18971259

[B66] Montenegro M. M. Ribeiro I. W. J. Kampits C. Saffi M. A. L. Furtado M. V. Polanczyk C. A. (2019). Randomized controlled trial of the effect of periodontal treatment on cardiovascular risk biomarkers in patients with stable coronary artery disease: preliminary findings of 3 months. *J. Clin. Periodontol.* 46 321–331. 10.1111/jcpe.13085 30761568

[B67] Moore W. E. (1987). Microbiology of periodontal disease. *J. Periodontal Res.* 22 335–341. 10.1111/j.1600-0765.1987.tb01595.x 2961863

[B68] Moreno S. Parra B. Botero J. E. Moreno F. Vasquez D. Fernandez H. (2017). Periodontal microbiota and microorganisms isolated from heart valves in patients undergoing valve replacement surgery in a clinic in Cali, Colombia. *Biomedica* 37 516–525. 10.7705/biomedica.v37i4.3232 29373772

[B69] Muniz F. W. Nogueira S. B. Mendes F. L. Rosing C. K. Moreira M. M. De Andrade G. M. (2015). The impact of antioxidant agents complimentary to periodontal therapy on oxidative stress and periodontal outcomes: a systematic review. *Arch. Oral. Biol.* 60 1203–1214. 10.1016/j.archoralbio.2015.05.007 26067357

[B70] Nakamura Y. Tagusari O. Seike Y. Ito Y. Saito K. Miyamoto R. (2011). Prevalence of periodontitis and optimal timing of dental treatment in patients undergoing heart valve surgery. *Interact. Cardiovasc. Thorac. Surg.* 12 696–700. 10.1510/icvts.2010.255943 21339340

[B71] Nakano K. Inaba H. Nomura R. Nemoto H. Takeda M. Yoshioka H. (2006). Detection of cariogenic *Streptococcus mutans* in extirpated heart valve and atheromatous plaque specimens. *J. Clin. Microbiol.* 44 3313–3317. 10.1128/jcm.00377-06 16954266 PMC1594668

[B72] Offenbacher S. Beck J. D. Moss K. Mendoza L. Paquette D. W. Barrow D. A. (2009). Results from the Periodontitis and Vascular Events (PAVE) Study: a pilot multicentered, randomized, controlled trial to study effects of periodontal therapy in a secondary prevention model of cardiovascular disease. *J. Periodontol.* 80 190–201. 10.1902/jop.2009.080007 19186958 PMC2778200

[B73] Ogrendik M. (2013). Rheumatoid arthritis is an autoimmune disease caused by periodontal pathogens. *Intl. J. Gen. Med.* 6 383–386. 10.2147/ijgm.s45929 23737674 PMC3668087

[B74] Okuda K. Ishihara K. Nakagawa T. Hirayama A. Inayama Y. (2001). Detection of *Treponema denticola* in atherosclerotic lesions. *J. Clin. Microbiol.* 39 1114–1117. 10.1128/jcm.39.3.1114-1117.2001 11230436 PMC87882

[B75] Ozcaka O. Bicakci N. Pussinen P. Sorsa T. Kose T. Buduneli N. (2011). Smoking and matrix metalloproteinases, neutrophil elastase and myeloperoxidase in chronic periodontitis. *Oral Dis.* 17 68–76. 10.1111/j.1601-0825.2010.01705.x 20646231

[B76] Pessi T. Karhunen V. Karjalainen P. P. Ylitalo A. Airaksinen J. K. Niemi M. (2013). Bacterial signatures in thrombus aspirates of patients with myocardial infarction. *Circulation* 127:e1-6. 10.1161/CIRCULATIONAHA.112.001254 23418311

[B77] Petersen P. E. Ogawa H. (2012). The global burden of periodontal disease: towards integration with chronic disease prevention and control. *Periodontology* 60 15–39. 10.1111/j.1600-0757.2011.00425.x 22909104

[B78] Ran S. Liu B. Gu S. Sun Z. Liang J. (2017). Analysis of the expression of NLRP3 and AIM2 in periapical lesions with apical periodontitis and microbial analysis outside the apical segment of teeth. *Arch. Oral. Biol.* 78 39–47. 10.1016/j.archoralbio.2017.02.006 28193569

[B79] Saito Y. Fujii R. Nakagawa K. I. Kuramitsu H. K. Okuda K. Ishihara K. (2008). Stimulation of *Fusobacterium nucleatum* biofilm formation by *Porphyromonas gingivalis*. *Oral Microbiol. Immunol.* 23 1–6. 10.1111/j.1399-302x.2007.00380.x 18173791

[B80] Sanz M. Marco Del, Castillo A. Jepsen S. Gonzalez-Juanatey J. R. D’aiuto F. (2020). Periodontitis and cardiovascular diseases: consensus report. *J. Clin. Periodontol.* 47 268–288. 32011025 10.1111/jcpe.13189 PMC7027895

[B81] Satoh M. Tabuchi T. Itoh T. Nakamura M. (2014). NLRP3 inflammasome activation in coronary artery disease: results from prospective and randomized study of treatment with atorvastatin or rosuvastatin. *Clin. Sci.* 126 233–241. 10.1042/cs20130043 23944632

[B82] Schultz-Haudt S. Bibby B. G. Bruce M. A. (1954). Tissue-destructive products of gingival bacteria from nonspecific gingivitis. *J. Dent. Res.* 33 624–631. 10.1177/00220345540330050601 13201695

[B83] Scott D. A. Krauss J. (2012). Neutrophils in periodontal inflammation. *Front. Oral Biol.* 15 56–83. 10.1159/000329672 22142957 PMC3335266

[B84] Sobaniec H. Sobaniec-Lotowska M. E. (2000). Morphological examinations of hard tissues of periodontium and evaluation of selected processes of lipid peroxidation in blood serum of rats in the course of experimental periodontitis. *Med. Sci. Monit.* 6 875–881. 11208425

[B85] Socransky S. S. Haffajee A. D. Cugini M. A. Smith C. (1998). Microbial complexes in subgingival plaque. *J. Clin. Periodontol.* 25 134–144. 10.1111/j.1600-051x.1998.tb02419.x 9495612

[B86] Song L. Dong G. Guo L. Graves D. T. (2018). The function of dendritic cells in modulating the host response. *Mol. Oral Microbiol.* 33 13–21. 10.1111/omi.12195 28845602 PMC5771978

[B87] Tang D. Shi Y. Kang R. Li T. Xiao W. Wang H. (2007). Hydrogen peroxide stimulates macrophages and monocytes to actively release HMGB1. *J. Leukoc. Biol.* 81 741–747. 10.1189/jlb.0806540 17135572 PMC1808495

[B88] Tomofuji T. Ekuni D. Yamanaka R. Kusano H. Azuma T. Sanbe T. (2007). Chronic administration of lipopolysaccharide and proteases induces periodontal inflammation and hepatic steatosis in rats. *J. Periodontol.* 78 1999–2006. 10.1902/jop.2007.070056 17916001

[B89] Tonetti M. S. (2009). Periodontitis and risk for atherosclerosis: an update on intervention trials. *J. Clin. Periodontol.* 36(Suppl. 10), 15–19. 10.1111/j.1600-051x.2009.01417.x 19432627

[B90] Tonetti M. S. D’aiuto F. Nibali L. Donald A. Storry C. Parkar M. (2007). Treatment of periodontitis and endothelial function. *N. Engl. J. Med.* 356 911–920. 17329698 10.1056/NEJMoa063186

[B91] Toregeani J. F. Nassar C. A. Nassar P. O. Toregeani K. M. Gonzatto G. K. Vendrame R. (2016). Evaluation of periodontitis treatment effects on carotid intima-media thickness and expression of laboratory markers related to atherosclerosis. *Gen. Dent.* 64 55–62. 26742169

[B92] Tsai C. C. Chen H. S. Chen S. L. Ho Y. P. Ho K. Y. Wu Y. M. (2005). Lipid peroxidation: a possible role in the induction and progression of chronic periodontitis. *J. Periodontal Res.* 40 378–384. 10.1111/j.1600-0765.2005.00818.x 16105090

[B93] Vidal F. Figueredo C. M. Cordovil I. Fischer R. G. (2009). Periodontal therapy reduces plasma levels of interleukin-6, C-reactive protein, and fibrinogen in patients with severe periodontitis and refractory arterial hypertension. *J. Periodontol.* 80 786–791. 10.1902/jop.2009.080471 19405832

[B94] Wang Y. Andrukhov O. Rausch-Fan X. (2017). Oxidative stress and antioxidant system in periodontitis. *Front. Physiol.* 8:910. 10.3389/fphys.2017.00910 PMC5693842 29180965

[B95] Wehmeyer M. M. Kshirsagar A. V. Barros S. P. Beck J. D. Moss K. L. Preisser J. S. (2013). A randomized controlled trial of intensive periodontal therapy on metabolic and inflammatory markers in patients With ESRD: results of an exploratory study. *Am. J. Kidney Dis.* 61 450–458. 10.1053/j.ajkd.2012.10.021 23261122 PMC3578050

[B96] White P. Cooper P. Milward M. Chapple I. (2014). Differential activation of neutrophil extracellular traps by specific periodontal bacteria. *Free Radic. Biol. Med.* 75(Suppl. 1):S53. 10.1016/j.freeradbiomed.2014.10.827 26461408

[B97] Xue F. Shu R. Xie Y. (2015). The expression of NLRP3, NLRP1 and AIM2 in the gingival tissue of periodontitis patients: RT-PCR study and immunohistochemistry. *Arch. Oral. Biol.* 60 948–958. 10.1016/j.archoralbio.2015.03.005 25841070

[B98] Yagi K. (1987). Lipid peroxides and human diseases. *Chem. Phys. Lipids* 45 337–351. 10.1016/0009-3084(87)90071-5 3319232

[B99] Yamaguchi Y. Kurita-Ochiai T. Kobayashi R. Suzuki T. Ando T. (2017). Regulation of the NLRP3 inflammasome in Porphyromonas gingivalis-accelerated periodontal disease. *Inflamm. Res.* 66 59–65. 10.1007/s00011-016-0992-4 27665233

[B100] Yanbaeva D. G. Dentener M. A. Creutzberg E. C. Wesseling G. Wouters E. F. (2007). Systemic effects of smoking. *Chest* 131 1557–1566. 10.1378/chest.06-2179 17494805

[B101] Zhou Q. B. Xia W. H. Ren J. Yu B. B. Tong X. Z. Chen Y. B. (2017). Effect of intensive periodontal therapy on blood pressure and endothelial microparticles in patients with prehypertension and periodontitis: a randomized controlled trial. *J. Periodontol.* 88 711–722. 10.1902/jop.2017.160447 28452620

